# Intracellular targeting of ascomycetous catalase-peroxidases (KatG1s)

**DOI:** 10.1007/s00203-013-0887-5

**Published:** 2013-04-16

**Authors:** Marcel Zámocký, Gerhard Sekot, Mária Bučková, Jana Godočíková, Christina Schäffer, Marián Farkašovský, Christian Obinger, Bystrík Polek

**Affiliations:** 1 Division of Biochemistry, Department of Chemistry, Vienna Institute of BioTechnology, University of Natural Resources and Life Sciences (BOKU), Muthgasse 18, 1190 Vienna, Austria; 2 Institute of Molecular Biology, Slovak Academy of Sciences, Dúbravská cesta 21, 84551 Bratislava, Slovakia; 3 Department of NanoBiotechnology, Vienna Institute of BioTechnology, University of Natural Resources and Life Sciences (BOKU), Muthgasse 11, 1190 Vienna, Austria

**Keywords:** Catalase-peroxidase (KatG), *Chaetomium*, Oxidative stress, Peroxisomal targeting, Thiolase, Real-time PCR

## Abstract

Bifunctional catalase-peroxidases (KatGs) are heme oxidoreductases widely spread among bacteria, archaea and among lower eukaryotes. In fungi, two KatG groups with different localization have evolved, intracellular (KatG1) and extracellular (KatG2) proteins. Here, the cloning, expression analysis and subcellular localization of two novel *katG1* genes from the soil fungi *Chaetomium globosum* and *Chaetomium cochliodes* are reported. Whereas, the metalloenzyme from *Ch. globosum* is expressed constitutively, *Ch. cochliodes* KatG1 reveals a slight increase in expression after induction of oxidative stress by cadmium ions and hydrogen peroxide. The intronless open reading frames of both Sordariomycetes *katG1* genes as well as of almost all fungal *katG1s* possess two peroxisomal targeting signals (PTS1 and PTS2). Peroxisomal localization of intracellular eukaryotic catalase-peroxidases was verified by organelle separation and immunofluorescence microscopy. Co-localization with the peroxisomal enzyme 3-ketoacyl-CoA-thiolase was demonstrated for KatGs from *Magnaporthe grisea*, *Chaetomium globosum* and *Chaetomium cochliodes*. The physiological role of fungal catalase-peroxidases is discussed.

**Electronic supplementary material:**

The online version of this article (doi:10.1007/s00203-013-0887-5) contains supplementary material, which is available to authorized users.

## Introduction

Catalase-peroxidases (mostly abbreviated as KatGs) represent a widespread protein family of heme *b* containing oxidoreductases (EC 1.11.1.21) capable of both efficient dismutation of hydrogen peroxide to oxygen and water as well as classical peroxidase activity with physiologically unknown endogenous electron donor(s) (Smulevich et al. 2006; Zamocky et al. 2008). These unique peroxidases evolved in ancestral bacteria revealing a complex gene-duplicated structure. Besides being found in numerous bacteria of all phyla, *katG* genes were also detected in genomes of lower eukaryotes, most prominently of sac and club fungi (Klotz and Loewen 2003; Zamocky and Obinger 2010). Phylogenetic analyses revealed that the corresponding gene was transferred during later steps of *katG* evolution via horizontal gene transfer (HGT) most probably from Bacteroidetes to ancestral fungal genomes (Zamocky et al. 2010, 2012a).

Genomic analysis demonstrated the occurrence of two gene paralogs coding for two distinct fungal catalase-peroxidases (KatG1 and KatG2) that differ in localization, structural and functional properties (Zamocky et al. 2009a, b). All KatG-containing fungi have an intracellular enzyme (KatG1), whereas, only phytopathogenic fungi have in addition a secreted (extracellular) species (KatG2) that seems to be involved in host attack (Zamocky et al. 2012b). Both the role and localization of eukaryotic KatG1 are unknown so far. Its *catalatic* activity suggests a role in H_2_O_2_ degradation, the latter being permanently produced as by-product of aerobic metabolism in the cells. Due to the presence of several (oxidative) catabolic pathways, peroxisomes that play a key role in redox signaling and lipid homeostasis (Gabaldón 2011; Schlüter et al. 2010) typically contain high amounts of monofunctional catalases (EC 1.11.1.6). Peroxisomes of filamentous fungi are rather scarcely investigated organelles, although their involvement in β-oxidation of fatty acids, oxidative stress response (Gabaldón 2011) and even in production of penicillin was already demonstrated (Kiel et al. 2000). Peroxisomes do not possess their own genetic apparatus, and all organelle proteins must be imported including monofunctional catalases. This was also demonstrated for yeast and fungal peroxisomes (Kragler et al. 1993). Recently, it was indicated that also bifunctional catalase-peroxidases could be imported into peroxisomes (Kiel et al. 2009).

In this contribution, we have analyzed the expression pattern and subcellular localization of three different intracellular ascomycetous catalase-peroxidases, namely KatG1s from *Magnaporthe grisea*, *Chaetomium globosum* and *Chaetomium cochliodes*, respectively. Cloning and expression analysis of *katG1* genes from the two soil fungi from the abundant family *Chaetomiaceae* are reported for the first time. Expression of catalase-peroxidases has been reported in related saprotrophic fungi *Podospora* *anserina* (Bourdais et al. 2012) and *Neurospora crassa* (Peraza and Hansberg 2002), but their subcellular localization has not been addressed so far. Analysis of the intracellular catalase-peroxidases clearly demonstrated the presence of two peroxisomal targeting signals (PTS1 and PTS2). Peroxisomal co-localization with the marker enzyme 3-ketoacyl-CoA-thiolase is demonstrated in this contribution by organelle separation as well as immunofluorescence microscopy. The physiological role of fungal KatG1s is discussed.

## Materials and methods

### Organisms and cultivation

*Chaetomium globosum* was obtained from cultivated isolates taken from medieval art objects deposited in Slovak National Gallery in Bratislava, Slovakia (Pangallo et al. 2009). *Chaetomium cochliodes* CCM F-232 was obtained from Czech Collection of Microorganisms at the Masaryk University, Faculty of Natural Sciences in Brno, Czech republic. *Magnaporthe grisea* (synonym *Pyricularia grisea*) was obtained from the culture collection of BOKU—University of Natural Resources and Life Sciences in Vienna, Austria. During experiments, all three mentioned fungi were maintained on potato dextrose (PD) medium (4 g potato extract, 20 g glucose/L pH 5.6) or on PD-agar plates (PD medium with 15 g agar/L) at 30 °C.

### Growth conditions and isolation of genomic DNA and mRNA

The three fungal strains were grown in 100-mL PD medium and incubated at 30 °C for 2–5 days with continuous shaking at 200 rpm. For probing the effect of oxidative stress on *katG1* expression, after 48 h of growth, the fungi were exposed to following agents for 1 h: hydrogen peroxide (0.01 M final), peroxyacetic acid (0.05 M final), paraquat (0.1 mM) and Cd^2+^ (5 mg/mL). Afterward the mycelia were separated from the broth by filtration through sterile filter paper (Whatman No. 1) and frozen at −80 °C for DNA and RNA extraction. The DNA was extracted with DNeasy Tissue Kit (QIAGEN, Hilden, Germany) and the RNA with Spectrum Plant Total RNA Kit (Sigma-Aldrich) according to the manufacturer’s instructions.

Total RNA (~3 μg) was reverse-transcribed in the presence of oligo(dT)_20_ primer in a \ volume of 20 μl by applying the Cloned AMV First-strand cDNA synthesis Kit (Invitrogen). Obtained product was used as template for RT-PCR. Primers CgkatGNterFWD + CgkatGintREV2 (Table 1) were used to study the level of expression of *katG* genes, and primers CgTIMfwd and CgTIMrev were used as a control of expression levels. For obtaining the complete sequences of *Ch. globosum* and *Ch. cochliodes* *katG1* genes, the primer pair CgkatGNtermFWD and CgtermREV was used. All PCR constructs were cloned in pCR-Blunt vector (Invitrogen). For quantification of the expression levels, real-time PCR was performed on 7900HT Fast Real-Time PCR System from Applied Biosystems with FastStart SYBR Green master mix (Roche) and special real-time PCR primers (Table 1). DNA sequencing of obtained RT-PCR clones and genomic PCR clones was performed at Comenius University Bratislava, Slovakia. Obtained novel DNA sequences were submitted to GenBank.

**Table 1:** List of DNA primers used in this work

Name of primer	Sequence in 5′ → 3′direction
CgkatGNterFWD	ATGGGTGAGTGCCCTGTCAACCATG
CgkatGintREV2	GAGATCAGCTTAGCAGGAGCAACGC
CgtermREV	CAACTTCGGTCCCGAAGATCCAGGC
CgtermrevB	CGTAACGGTCGAGGTTCATCACCTTG
CgkatGrtFWD1	CTCTTATGGCCTGTCAAGCA
CgkatGrtREV1	AAGCCAAACGTCTTGAAACC
CgTIMfwd	GAACCAAGGAGTCCATCAAGAG
CgTIMrev	ATGACGATCTTCTTCCAGTCGT
CgTIMRTrev	CTTGCGCAAGCTGGAGGTAGAG

Electrophoresis of isolated genomic DNA and of cDNA clones was performed in 1.0 % agarose run in TAE buffer. For staining of DNA bands, GoldView (SBS Genetech) was used.

### Homogenization of fungal cultures and measurement of enzymatic activity

Mechanical disruption of frozen fungal mycelia was performed in 50 mM sodium phosphate, 2 mM EDTA (pH 8.2) at 4 °C with glass beads (diameter 0.4 μm). The homogenates were centrifuged at 15,000 g for 10 min. Supernatants were used for the determination of enzymatic activity and protein concentration. Catalase activity was determined spectrophotometrically at pH 7.0 by monitoring the decomposition of H_2_O_2_ at 240 nm with an extinction coefficient of 43.6 M^−1^ cm^−1^. One unit of catalase activity (1 U) was defined as the amount of enzyme that catalyzes the decomposition of 1 μmol of H_2_O_2_ per min (Roggenkamp et al. 1974). Peroxidase was assayed spectrophotometrically at pH 5.5 by monitoring the oxidation of *o*-dianisidine dihydrochloride at 460 nm (ε_460 nm_ = 11.3 × 10^3^ M^−1^ cm^−1^). One unit of peroxide activity (1 U) was defined as the amount of activity that produces 1 μmol of oxidized *o*-dianisidine per min (Zou and Schrempf 2000). Total soluble protein in crude extracts was quantified using a Coomassie stain-based assay (Pierce, Rockford, IL). All measurements in this study were performed at room temperature in triplicates and analyzed individually. An average standard deviation was calculated. The bars shown in the figures indicate a standard error of the mean (*n* = 3).

### Isolation of fungal peroxisomes

The method for isolation of fungal peroxisomes originally developed for *Penicillium chrysogenum* (Kiel et al. 2009) was used with following modifications. The filaments of the three fungi (grown in PD medium) were filtered using a Whatman filter paper No.1 and washed with a KC buffer (0.8 M KCl, 10 mM sodium citrate pH 6.2). They were further protoplasted for 3 h at 25 °C in KC buffer containing 20 mg/mL lysing enzymes from *Trichoderma* (Sigma-Aldrich). The protoplasts were consecutively washed in KC buffer with 1 mM PMSF, with a mixture of 50 % KC buffer and 50 % sorbitol buffer (1.2 M sorbitol and 1 mM PMSF) and, finally, with 100 % sorbitol buffer containing 1 mM PMSF. Obtained samples were subjected to differential centrifugation comprising three consecutive steps (4 °C): 10 min at 4,000 g, 10 min at 6,000 g and 20 min at 30,000 g, respectively. The pellet from the last centrifugation step was resuspended in ice-cold 40 % saccharose in B buffer (5 mM MES 1 mM MgCl_2_, 1 mM EDTA, pH 5.5) and applied to ultracentrifugation at 32,000 g for 2.5 h. Up to 20 fractions with the size of 1,5 ml were obtained from each gradient. These fractions were precipitated with equal volume of 50 % TCA and frozen at −20 °C overnight. After 5-min centrifugation at 20,000g, resulting pellets were resuspended in a minimal volume of a solution containing 1 % SDS and 0.1 M NaOH for protein electrophoresis.

### Protein electrophoresis and Western blot

Protein electrophoresis of sucrose gradient fractions under denaturing conditions was performed in Mini-Protean Tetra Cell apparatus from BioRad. Ten percentage SDS gels were either stained with Coomassie Brilliant Blue or were used for Western blotting. Western blots were run in Mini Trans-blot cells from BioRad. KatG-specific bands were detected with a polyclonal antibody raised against *Synechocystis* KatG, as described previously (Zamocky et al. 2012b). Detection of peroxisomal-specific fractions of sucrose gradient was monitored with anti-Fox3 antibody raised against the peroxisomal enzyme 3-ketoacyl-CoA-thiolase from yeast (in the same manner as for anti-KatG antibody).

Native polyacrylamide gels with a linear gradient of acrylamide (4–18 %) were stained for either catalase or peroxidase activity. Catalase activity was visualized after a brief incubation in 0.003 % hydrogen peroxide followed by addition of a freshly prepared mixture of 2 % potassium ferricyanide and 2 % ferric chloride (Woodbury et al. 1971). Peroxidase activity was visualized with 3,3΄-diaminobenzidine tetrahydrochloride (DAB) using the method described by Wayne and Diaz 1986.

### Immunofluorescence microscopy

Localization of expressed KatG1 from *Chaetomium globosum*, *Chaetomium cochliodes* and *Magnaporthe grisea* was tested by means of immunofluorescence staining with concomitant fluorescence microscopy. For this purpose, the three fungal strains were cultivated on PD-agar plates. Besides the fungal samples grown aerobically with no additives, positive controls were generated by induction of peroxisome proliferation with the addition of 0.2 ml olive oil/plate for 1 h. Anaerobically grown fungi incubated in the Anaerocult kit (Merck chemicals), served as peroxisome-negative controls.

Anti-KatG-antisera were tested by Western blot analysis for specificity prior to labeling experiments. Oleic acid-induced positive controls were washed once with pure acetone and together with the other samples were fixed in pure ethanol for 15 min at −70 °C. Samples were washed once with PBS (phosphate buffered saline) buffer, and unspecific binding sites were blocked (1 h, 25 °C, constant stirring) in PBS containing 2 % BSA. Subsequently to another washing step with PBS, the samples were exposed to primary polyclonal rabbit anti-αFox3 antiserum (gift from Prof. Kunau, Bochum Germany) diluted 1:100 with PBS (1 h, 25 °C, constant stirring). After removal of unspecific binders by washing the fungal samples three times with tris-buffered saline containing 1 % Tween-20 (TBST), the secondary anti-rabbit TRITC (tetramethyl rhodamine isothiocyanate) conjugate antibody (Sigma-Aldrich) was diluted to a final concentration of 0.03–0.06 mg/mL with PBS and added to the samples, followed by exposure for 1 h at 25 °C under constant stirring. Unbound antibodies were removed by washing three times with 1 ml TBST, and the samples were further labeled with anti-SynKatG (Agrisera)-FITC-conjugated antibody (1 h, 25 °C, constant stirring). In detail, 1 mg of lyophilized anti-KatG antibody was resuspended in 500 μl of 0.05 M borate buffer and labeled with Pierce^®^ FITC (fluorescein isothiocyanate) Antibody Labelling Kit (Thermo Scientific) according to the manufacturer’s instructions. Unbound antibodies were removed by three washing steps with TBST and one washing step with PBS. Analysis of the samples was performed with an Eclipse TE2000-S fluorescence microscope (Nikon, Vienna, Austria). Pictures were taken with a DS-Qi1 Mc-camera (Nikon), and the same image section was recorded in bright field, as well as in the FITC and the TRITC channel, the respective overlay was generated with NIS Elements BR software (Nikon).

### Sequence alignment and analysis of peroxisomal targeting signals

Multiple sequence alignment of protein sequences was conducted with ClustalX (Larkin et al. 2007). KatG-optimized parameters were as follows: for pairwise alignment gap opening penalty 9, gap extension penalty 0.15, and for multiple alignment gap opening penalty 8, gap extension penalty 0.2. Gonet protein weight matrix was applied, and the gap-separation distance was set to 4. Peroxisomal targeting signals were predicted in the obtained KatG protein sequences with two prediction programs. For the presence of peroxisomal targeting signal 1 on the C-termini, the PTS1 predictor (Neuberger et al. 2003) was used with fungi-specific function. For the occurrence of peroxisomal targeting signal 2 near the N-termini, the prediction algorithm for the presence of a canonical motif within an amphipathic helix (Kunze et al. 2011) was used. The secondary structure of KatGs was predicted with PSIPRED method version 3.0 (McGuffin et al. 2000).

## Results and discussion

Recently, the first eukaryotic intracellular catalase-peroxidase (KatG1) from the rice blast fungus *Magnaporthe grisea* was cloned and heterologously expressed in *E. coli*. Its biochemical properties were shown to be similar to those determined for prokaryotic KatGs (Zamocky et al. 2009a). However, several questions remained open including subcellular localization and physiological relevance. In order to clarify these issues in general for the group of KatG1 proteins, we have additionally cloned two novel *katG* genes from important soil-borne fungi (*Ch. globosum* and *Ch. cochliodes*) and compared them with the already known enzyme from *M. grisea*. Table 2 lists all accession numbers of gene & protein sequences made available within this work.

**Table 2:** NCBI accession numbers of clones investigated in this work

NCBI entry	Description
JF937065	*Chaetomium cochliodes katG1* DNA-complete
AEQ54765	*Chaetomium cochliodes* KatG1 protein-complete
CH408031.1	*Chaetomium globosum* genomic DNA with *katG1*
EAQ88655	*Chaetomium globosum* KatG1 protein-complete
CH476831.1	*Magnaporthe grisea* genomic DNA with *katG1*
EDJ99644	*Magnaporthe grisea* KatG1 protein-complete

**Fig. 1:**
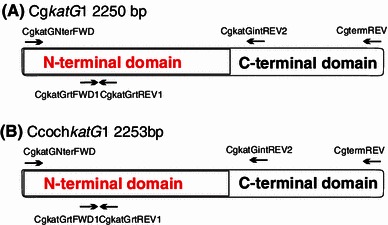
Schematic presentation of two *katG*1 genes from *Chaetomiaceae* analyzed in this work. The division of coding regions in two major parts coding for two distinct domains is indicated as well as location of all primers used for PCR

**Fig. 2:**
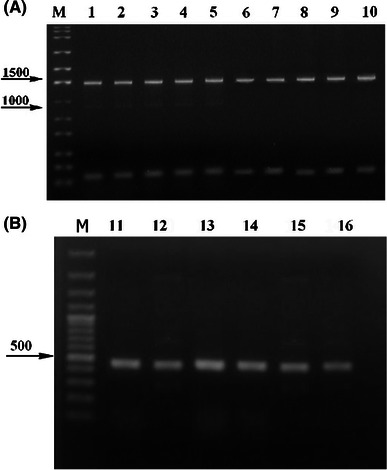
DNA gel electrophoresis of various *Chaetomium* samples. **a** DNA electrophoresis in 1 % agarose of RT-PCR products isolated from *Ch. globosum* grown under standard conditions (*lane 1*, PD medium with no oxidative stress) or oxidative stress induced by addition of 0.1 mM paraquat (*lane 2*), or 0.05 M peroxyacetic acid (*lane 3*), or 5 mg/mL Cd^2+^ (*lane 4*), or 10 mM H_2_O_2_ (*lane 5*). In *lanes 1*–*5,* the same reaction volume of RT-PCR product from *Ch. globosum* was loaded for comparison. Products isolated from *Ch. cochliodes* grown under standard conditions (*lane 6*) or oxidative stress induced by addition of 0.1 mM paraquat (*lane 7*) or 0.05 M peroxyacetic acid (*lane 8*), or 5 mg/ml Cd^2+^ (*lane 9*), or 10 mM H_2_O_2_ (*lane 10*). In *lanes 6*–*10,* the same reaction volume of RT-PCR product was loaded. For all samples loaded in *lanes 1*–*10,* the combination of primers CgkatGNterFWD & CgkatGintREV2 was used for amplification (Table 1). B) DNA electrophoresis in 1 % agarose of control RT-PCR products from *Ch. cochliodes* (*lanes 11*–*13*) and *Ch. globosum* (*lanes 14*–*16*). The same reaction volume of RT-PCR product was loaded in *lanes 11*–*16* in different growth phases: *lanes 11* and *14* after 1 day of cultivation, *lanes 12* and *15* after 2 days of cultivation and *lanes 13* and *16* after 3 days of cultivation; For all samples loaded in *lanes 11*–*16* the combination of primers CgTIMfwd & CgTIMrev was used for amplification (Table 1). *Lanes M* molecular mass standards with size given in base pairs (bp)

**Fig. 3:**
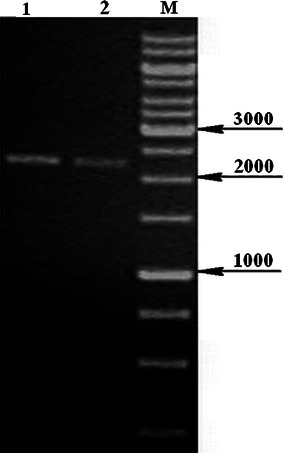
DNA electrophoresis in 1 % agarose of PCR products from genomic DNA of *Ch. globosum* (*lane 1*) and *Ch. cochliodes* CCM F232 (*lane 2*) containing whole *katG* genes; *lane M* molecular mass standard with size given in base pairs (bp)

The 2,250-bp-long genomic sequence of *Chaetomium globosum* strain CBS 148.51 (sequenced at Broad institute in USA, genomic scaffold_3) contains the complete *katG*1 ORF, and translation of this DNA sequence into corresponding amino acid sequence reveals that there are no introns. We have completely sequenced the homologous gene from *Ch. cochliodes* and found also no introns after translation. Schematic presentation of both genes with location of primers is presented in Fig. 1. RT-PCR (Fig. 2) yielded a gene fragment of cDNA of the same size that would be expected in the genomic DNA. The size of PCR products from genomic DNA for the entire coding region (Fig. 3, 2,250 bp) confirms the intronless state of *katG1* in *Ch. globosum* and *Ch. cochliodes*. This is typical for almost all sequences from the KatG1 group and underlines the proposed HGT event from Bacteroidetes to ancestral fungal genomes (Zamocky et al. 2012a, b). The new 2,253-bp coding sequence from yet undescribed sac fungus *Ch. cochliodes* differs only marginally from the genomic sequence of *Ch. globosum* (91 % identity, 95 % overall homology and 1 gap in the whole ORF). However, these *katG1* coding regions reveal a higher overall variability than, for example, the ITS1 noncoding DNA regions routinely used for fungal taxonomical purposes where there is up to 99 % identity (within a 500-bp region) between the two soil fungi. In contrast to *Chaetomia* genomes, the corresponding genomic region of KatG1 from *M. grisea* contains one intron near its 3′-terminus dividing the region coding for one of the peroxisomal targeting sequences (PTS1) from the remaining part of the gene (see below).

In the next step, we have analyzed *katG1*-mRNA occurrence and KatG1 expression pattern of the soil fungi. RT-PCR data clearly demonstrate a constitutive mode of expression for *katG1* gene in both *Ch. globosum* and *Ch. cochliodes*. Upon induction of oxidative stress by hydrogen peroxide, peroxyacetic acid, and paraquat or Cd^2+^, no significant increase above the basal expression level was observed (Fig. 2a). This was compared with parallel control RT-PCRs for both *Chaetomia* based on TIM primers (Fig. 2b) coding for constitutively expressed triose phosphate isomerase from glycolysis (Table 1). Real-time PCR results (Table 3) revealed a constitutive level of *katG1*-mRNA expression for *Ch. globosum,* and for *Ch. cochliodes,* there was a small increase in cadmium samples (typical example is presented in Supplemental Fig. 1). Obtained results are in good agreement with previous analyses performed under comparative conditions for KatG from *M. grisea* (Zamocky et al. 2009a), where only the induction of mycelia with PQ also led to a slight increase in *katG1*-mRNA expression.

**Table 3:** Quantification of *katG1* expression with real-time PCR using internal standard dilutions series (an internal fragment of *C. globosum katG1* gene, 1012 bp long cloned in plasmid pRb3)

Sample	C_t_ value	c_init_ DNA [ng/μl]
Ch. globosum control	31.81	26.2
Ch. globosum H_2_O_2_	32.32	25.7
Ch. globosum PAA	32.01	26.0
Ch. globosum PQ	32.55	25.5
Ch. globosum Cd^2+^	32.48	25.6
Ch. cochliodes control	43.91	18.1
Ch. cochliodes H_2_O_2_	43.09	18.5
Ch. cochliodes PAA	44.50	17.8
Ch. cochliodes PQ	48.94	16.0
Ch. cochliodes Cd^2+^	40.70	19.7

For each sample, average values of triplicate determinations are given

**Fig. 4:**
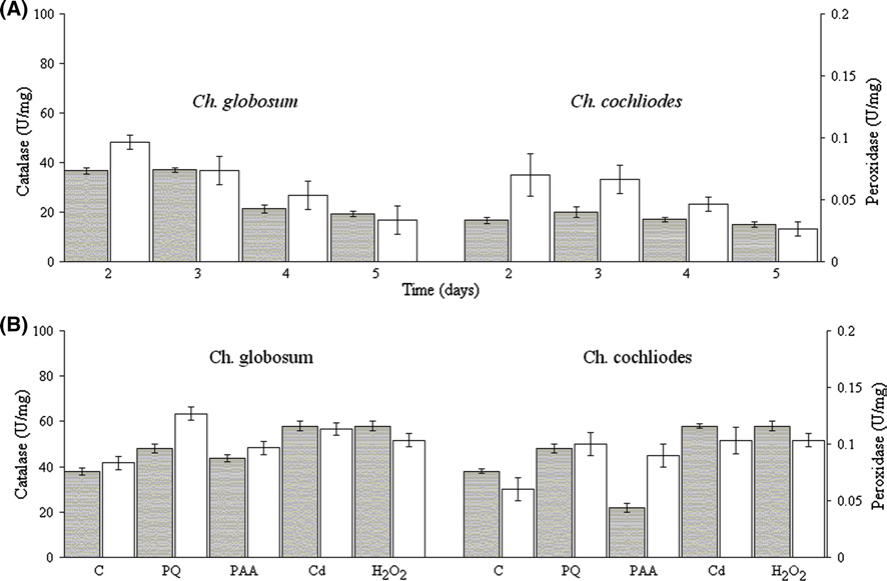
Catalatic (*gray columns*) and peroxidatic activities (*empty columns*) in crude extracts of *Ch. globosum* and *Ch. cochliodes* grown in PD medium. **a** Specific activities in various phases of growth at 30 °C (noninduced samples). **b** Specific activities in the exponential phase of growth with applied stress inductors incubated for 1 h at 30 °C. All presented data are mean values ± SD of three replicates

**Fig. 5:**
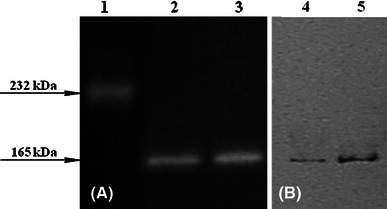
Catalase (**a**) and peroxidase (**b**) activity staining in 4–18 % native polyacrylamide gradient gel for *Ch. globosum* (*lane 2* and *4*) and *Ch. cochliodes* CCM F232 (*lane 3 and 5*). Bovine liver catalase (232 kDa) was used as a monofunctional tetrameric catalase standard (*Lane 1*)

We have further analyzed the expression pattern for intracellular catalase and peroxidase activities in crude extracts of *Ch. globosum* and *Ch. cochliodes* (Fig. 4). Recorded activities were highest in the exponential phase of growth (Fig. 4a) in both cases. The highest level of specific activities in samples induced with paraquat could be detected for *Ch. globosum* (Fig. 4b). For *Ch. cochliodes,* the highest specific activities were obtained for cadmium- and hydrogen peroxide-induced samples (Fig. 4b). Apparently, intracellular catalase-peroxidases are produced constitutively in the fast aerobically growing mycelia, ready for decomposition of the cellular ROS. The presence of an oxidative stressor further increases their production but only to a slightly higher level. Native gradient protein electrophoresis (Fig. 5) demonstrated the typical bifunctionality of these new eukaryotic KatG1s. The same protein band of a homodimer (calculated MW of around 165 kDa) stained positively by inhibiting the formation of Berlin blue in catalase assay (Fig. 5a) as well as oxidizing DAB in peroxidase assay (Fig. 5b).

**Fig. 6:**
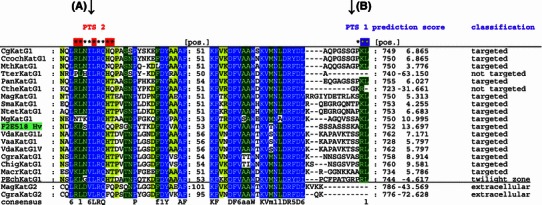
Protein multiple sequence alignment showing the presence (or absence) of PTS1 and PTS2 signals in 20 selected KatG sequences. *Color* scheme in columns: *blue*, highest level of conservation; *green*, intermediate level of conservation; *yellow*, low level of conservation. *Left side* region around PTS2 near the N-terminus of KatG proteins. *Right side* region on the C-terminus with PTS1 comprising last 12 amino acids. For complete alignment see Supplemental Figure 2

Furthermore, we probed the subcellular localization of eukaryotic KatG1 starting with gene analysis. Multiple sequence alignment of 20 full-length sequences coding for (ascomycetous) catalase-peroxidases (complete in Suppl. Fig. 2) demonstrated the presence of two peroxisomal targeting signals (PTS) predicted by two independent algorithms (Fig. 6). For comparison, we have included also two extracellular KatG2 sequences (lower sequence pair) that cannot be imported in the peroxisomes. From remaining 18 KatG1 sequences, only *Penicilium chrysogenum* catalase-peroxidase falls in the “twilight zone” due to PTS1 prediction, and all others are clearly classified. However, this KatG was already suggested to be located in peroxisomes due to evidence from LC–MS/MS (to 32 % coverage) (Kiel et al. 2009). Very interestingly, the KatG1 sequences from two thermophilic fungi (*Thielavia terrestris* and *Chaetomium thermophilum*) do not fulfill the criteria for peroxisome import due to lack of PTS1 signal (Fig. 6b), but the import of at least KatG1 from *Ch. thermophilum* cannot be ruled out according to a rather high conservation of the canonical PTS2 motif (Fig. 6a). The third presented KatG1 from a thermophilic fungus, MthKatG1 possesses both PTS1 and PTS2 signals, but the subcellular location of these technologically interesting oxidoreductases needs to be verified experimentally. All remaining sequences of KatG1s from mesophilic organisms, mainly from phytopathogenic fungi revealed a high level of conservation of both PTS signals. Most intriguingly, the translated cDNA sequence from *Hordeum vulgare* (Matsumoto et al. 2011) reveals the highest prediction score for fungal PTS1 (Fig. 6b) and PTS2 (Fig. 6a). Thus, it is tempting to speculate whether this sequence originates from *Hordeum vulgare* or from its yet unknown fungal parasite (also the remaining parts of the sequence resemble fungal KatG1—Supplemental Fig. 2). The sequences of the three KatGs investigated in this work revealed a good level of PTS1 and PTS2 conservation (Fig. 6a, b) and thus, must be considered as targeted into peroxisomes.

**Fig. 7:**
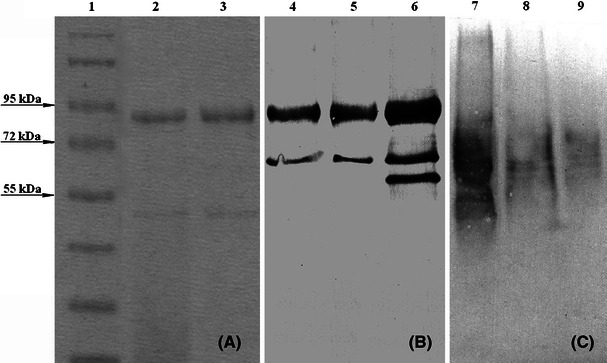
SDS-PAGE (10 %) and Western blot of peroxisomal fractions (from sucrose gradient centrifugation) of *Chaetomium* samples. **a** Coomassie brilliant blue stain; **b** Western blot with anti-KatG antibody; **c** Western blot with anti-Fox3 antibody. Lanes: 1—molecular weight standard (Fermentas), 2—*Ch. cochliodes* CCMF232 gradient fraction 5–6, 3—*Ch.* *globosum* gradient fraction 5–6, 4 - *Ch. cochliodes* CCMF232 gradient fraction 5-6, 5 – *Ch.* *globosum* gradient fraction 5-6, 6 – *M. grisea* affinity purified KatG1, 7 – *Ch. globosum* gradient fraction 5-6, 8 – fraction 7-8, 9 – fraction 9-10 of the same sucrose gradient as in lane 3

These findings prompted us to verify the in vivo localization of the three ascomycetous KatG1s experimentally. Crude extracts of *Ch. globosum* and *Ch. cochliodes* were subjected to differential centrifugation and consecutively to sucrose gradient ultracentrifugation. Sucrose gradient fractions were TCA-precipitated (Kiel et al. 2009) loaded on SDS-PAGE and analyzed by Western blot (Fig. 7). In order to verify peroxisomal localization, both KatG and the peroxisomal marker protein 3-ketoacyl-CoA-thiolase (EC 2.3.1.16) (Goh et al. 2011; Lee et al. 2009) were probed by polyclonal anti-KatG (Fig. 7b) and anti-Fox3 antibodies (Fig. 7c), respectively. With both organisms (*Ch. globosum* and *Ch. cochliodes*), the two antibodies were bound to proteins from the same fraction at ~83 kDa (KatG) and ~59 kDa (molar mass of peroxisomal thiolase subunit according to UniProt Entry G3MEU1) (Fig. 7b, c).

**Fig. 8:**
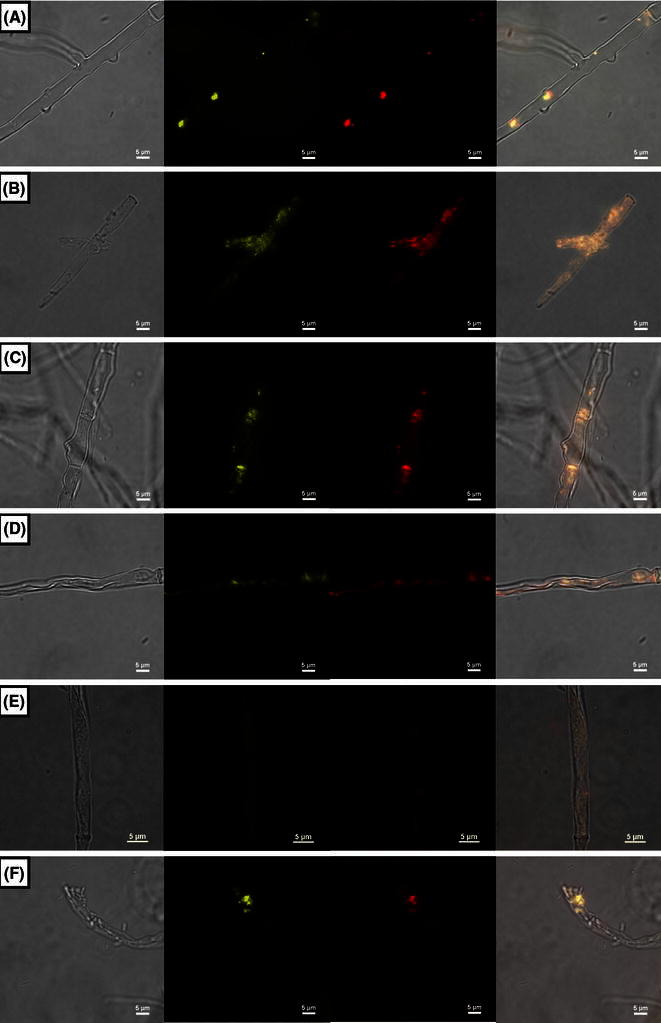
Co-localization of KatG and 3-ketoacyl-CoA thiolase detected with immunofluorescence microscopy. A, B – *Magnaporthe grisea*; C,D – *Chaetomium cochliodes*; E,F- *Chaetomium globosum*. A,C,E – noninduced samples; B,D,F – samples after induction with oleic acid. Presentation of panels from left to right: bright field (no fluorescence), FITC channel, TRITC channel, merged picture, respectively

Furthermore, the co-localization of thiolase and KatG1 in peroxisomes of fungi could be confirmed by immunofluorescence microscopy and double-staining for localization of KatG1 and thiolase (Fig. 8). Additionally, we tested induction of formation of peroxisomes by addition of 0.1 % (v/v) oleic acid. Here a different behavior of *Magnaporthe* and *Chaetomia* in various growth media could be observed. Whereas, for *Magnaporthe* *grisea,* a clear difference between noninduced and oleic acid-induced sample is obvious (Fig. 8a, b), in case of *Ch. cochliodes* and *Ch. globosum,* no increase in the number of peroxisomes occurred (Fig. 8c–e). This is also consistent with recorded activity profiles (Fig. 4b and (Zamocky et al. 2009a). Increased formation of peroxisomes and their matrix proteins in phytopathogenic *M. grisea* in oleic acid-enriched medium could be explained by direct involvement of a lipid component (i.e., glycerol) in the first steps of pathogenic invasion in rice tissue, namely during formation of the specialized infection structure, the appressorium (Caracuel-Rios and Talbot 2007; Goh et al. 2011).

For MagKatG1 the co-localization data also support the presence of a short intron predicted on the C-terminus, which is rare for fungal *katG1* genes. The PTS1 signal is located on the short exon 2. Previous biochemical characterization (Zamocky et al. 2009a) was performed without C-terminal exon 2, suggesting that it has no impact on the overall catalase and peroxidases activity of the purified protein.

Summing up, intracellular fungal catalase-peroxidases (KatG1s) are constitutively expressed and even upon addition of oxidative stressors, the mRNA concentration of *katG1* as well as the bifunctional activity remain at a constant level. In prokaryotes, KatGs are located in the cytosol, whereas, in eukaryotic organisms, two signals target these bifunctional metalloenzymes to peroxisomes. This was so far described for monofunctional (typical) catalases that often represent the major protein in peroxisomes. Their apparent role is efficient decomposition of hydrogen peroxide (Ozimek et al. 2005; Pollegioni et al. 2007). Most probably, KatG1 plays a similar role because of its unique and efficient *catalatic* activity. Fungi are known to have a broad armory of enzymes especially in oxidative defence reactions and thus some fungi apparently have both catalases and catalase-peroxidases located in peroxisomes for H_2_O_2_ degradation. Remains the question of the role of the relatively unspecific peroxidase activity of KatG1 (Smulevich et al. 2006; Zamocky et al. 2008). Generally, there is no significant difference between prokaryotic and eukaryotic KatGs, suggesting that the putative endogenous one-electron donor is not a metabolite specific for a distinct prokaryotic or eukaryotic metabolic pathway.

## Electronic supplementary material

Below is the link to the electronic supplementary material.

A typical profile of real-time PCR obtained with 7900HT Fast Real-Time PCR System from Applied Biosystems. Combination of primers CgkatGrtFWD1 & CgkatGrtREV1 was used for these samples (PDF 1838 kb)

Complete multiple sequence alignment of twenty ascomycetous catalase-peroxidases (KatGs) obtained with ClustalX. Color scheme is the same as in Fig. 6 (PDF 131 kb)

## Abbreviations

BSA: Bovine serum albumin
*Ch. globosum*: *Chaetomium globosum*
*Ch. cochliodes*: *Chaetomium cochliodes*
DAB: 3,3′-diaminobenzidine tetrachloride
Fox3: Yeast 3-ketoacyl-CoA-thiolase
HGT: Horizontal gene transfer
KatG1: Intracellular catalase-peroxidase
KatG2: Secreted (extracellular) catalase-peroxidase
*M. grisea*: *Magnaporthe grisea*
ORF: Open reading frame
PAA: Peroxyacetic acid
PMSF: Phenylmethyl sulfonyl fluoride
PQ: Paraquat
PTS1: Peroxisomal targeting signal 1
PTS2: Peroxisomal targeting signal 2
TCA: Trichloroacetic acid
