# Supplementary figures and images for: Intracellular targeting of ascomycetous catalase-peroxidases (KatG1s)
Source: Arch Microbiol. 2013 Apr 16;195(6):393–402. doi: 10.1007/s00203-013-0887-5 (PMC3668122; doi:10.1007/s00203-013-0887-5)

# *Ch. globosum* cDNA from PQ sample

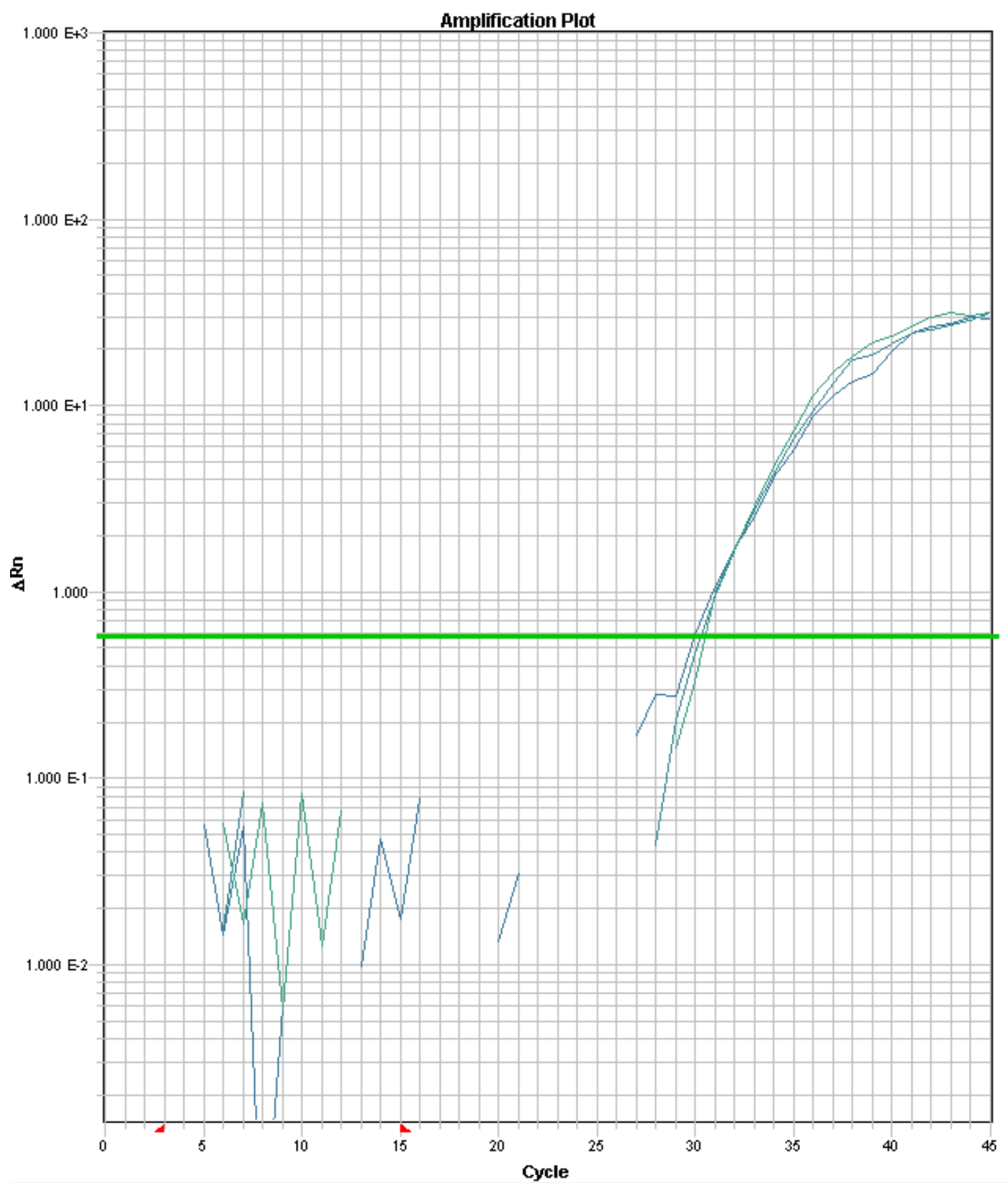

Supplement: Supplementary file 1 — A typical profile of real-time PCR obtained with 7900HT Fast Real-Time PCR System from Applied Biosystems. Combination of primers CgkatGrtFWD1 & CgkatGrtREV1 was used for these samples (PDF 1838 kb) [file 203_2013_887_MOESM1_ESM.pdf]
