# Supplementary material for: Intracellular targeting of ascomycetous catalase-peroxidases (KatG1s)
Source: Arch Microbiol. 2013 Apr 16;195(6):393–402. doi: 10.1007/s00203-013-0887-5 (PMC3668122; doi:10.1007/s00203-013-0887-5)
Supplement: Supplementary file 2 — Complete multiple sequence alignment of twenty ascomycetous catalase-peroxidases (KatGs) obtained with ClustalX. Color scheme is the same as in Fig. 6 (PDF 131 kb) [file 203_2013_887_MOESM2_ESM.pdf]

```
                      *        20         *        40         *        60         *        80         *       100
CgKatG1    : ---------------------------------------------MGE----CP-VNH---------------------------ANVAGGGTRNNDWWP :  23
CcochKatG1 : ---------------------------------------------MGE----CP-VNR---------------------------ANVAGGGTRNNDWWP :  23
MthKatG1   : ---------------------------------------------MGE----CP-VRHS--------------------------ANVAGGGTRNTDWWP :  24
TterKatG1  : ---------------------------------------------MGE----CP-NRF---------------------------ANAAGGGTRNTDWWP :  23
PanKatG1   : ---------------------------------------------MGE----CP-VNHTKS------------------------ANVAGGGTRNIDWWP :  26
CtheKatG1  : ---------------------------------------------MGE----CP-VRH---------------------------DNVAGGGTRNTDWWP :  23
MagKatG1   : ---------------------------------------------MGE----CP-LRT---------------------------ANVAGGGTRNRDWWP :  23
SmaKatG1   : ---------------------------------------------MSE----CP-VRK---------------------------SNVGGGGTRNNDWWP :  23
NtetKatG1  : ---------------------------------------------MSE----CP-VRK---------------------------SNVGGGGTRNHDWWP :  23
MgKatG1    : ---------------------------------------------MS--ANGCP-IKL---------------------------ANVGGGGTRNRDWWP :  25
F2E518_Hv  : ---------------------------------------------MGE----CP-VDHKN-------------------------LNIGGGGTRNTDWWP :  25
VdaKatG1L  : ---------------------------------------------MAESESKCP-VHQLN--------------------------NVGGGGTRNRDWWP :  28
VaaKatG1   : ---------------------------------------------MAESESKCP-VHKLN--------------------------NVGGGGTRNRDWWP :  28
VdaKatG1V  : ---------------------------------------------MAESESKCP-VHRLN--------------------------NVGGGGTRNRDWWP :  28
CgraKatG1  : ---------------------------------------------MAEE--KCPFIRTM---------------------------NTGGGGTKNRDWWP :  26
ChigKatG1  : ---------------------------------------------MAED--KCPFIRTM---------------------------NTGGGXTKXRDWWP :  26
MacrKatG1  : ---------------------------------------------MAES--KCPHL------------------------------NVAGGGTRNRDWWP :  23
PEchKatG1  : ---------------------------------------------MSE----CP-VAHKR-------------------------SNVAGGGTRNTDWWP :  25
MagKatG2   : ---------------------------MHASLSSWLLAASLLTQPISVSGQGCPFAKRDGTVDSSLPQKRADAPETTTFGRCAVKSNQAGGGTRSHDWWP :  73
CgraKatG2  : ---------------------------MHAKLSNCLLAASAI-L-PSVSAQGCPFAKRDGSVSSELPKR----DTAATFGRCPTISNEAGGGTRSRDWWP :  67
                                                              e    CP                                N  GGgT4n DWWP
```

Zamocky et al., Supplem. Fig. 2

PTS 2

```
                        *        120         *        140         *        160         *        180         *        200
CgKatG1     : NQLRLNILRQHQPASSPYSKEFDYAAAFKSLDYEALKKDITAVMTDSQDWWPADFGHYGGLFIRMAWHSAGTYRVFDGRGGGSQGQQRFAPLNSWPDNVS : 123
CcochKatG1  : NQLRLNILRQHQPASNPYNKEFDYAAAFKSLDYDALKKDIAAVMTDSQDWWPADFGHYGGLFIRMAWHSAGTYRVFDGRGGGSQGQQRFAPLNSWPDNVS : 123
MthKatG1    : NQLRLNILRQNQPASNPQ-KDLDYAAAFKSLDYEGLKKDLRALMTDSQDWWPADFGHYGGLFIRMAWHSAGTYRVFDGRGGGSQGQQRFAPLNSWPDNVS : 123
TterKatG1   : NRLPLHILRQHQPASSPY-KDFDYRAAFKSLDYDALKQDLRALMTDSQDWWPADFGHYGGLFIRMAWHSAGTYRVFDGRGGGGQGQQRFAPLNSWPDNVS : 122
PanKatG1    : NQLRLNILRQHTAASDPFHKEFNYAAAFKSLDYNALKKDLTDLMTNSQDWWPADFGHYGGLFIRMAWHSAGTYRVFDGRGGGGQGQQRFAPLNSWPDNVS : 126
CtheKatG1   : KQLRLNILRQHQPASNPYPKDFDYAAAFKSLDYEALKQDIKAVLTDSQDWWPADFGHYGGLFIRMAWHSAGTYRVFDGRGGGGQGQQRFAPLNSWPDNVS : 123
MagKatG1    : NTLKLNILRQHTEATNPYDPNFDYAEAFKSLDYEGLKKDLRALMTDSQEYWPADFGHYGGLFVRMAWHSAGTYRVMDGRGGGGQGQQRFAPLNSWPDNVS : 123
SmaKatG1    : EQLKLNILRQHTPVTNPLDKDFDYAAAFKSLDYEGLKKDLTSLMTDSKDWWPADFGHYGGLFIRMAWHSAGTYRVMDGRGGGGEGQQRFAPLNSWPDNVS : 123
NtetKatG1   : AQLRLNILRQHTPVSNPLDKDFDYAAAFKSLDYEGLKKDLTKLMTDSQDWWPADFGHYGGLFIRMAWHSAGTYRVTDGRGGGGEGQQRFAPLNSWPDNVS : 123
MgKatG1     : NELNTKILRQHTAATDPFGKQFDYPAAFKSLDYNGLKKDLNDLMTDSKDFWPADFGHYGGFFVRMAWHSAGTYRVADGRGGGGEGQQRFAPLNSWPDNAN : 125
F2E518_Hv   : NDLKLSILRQQQPSGNPYTKDFDYVAAFKSLDYDALKKDLTALMTDSQEWWPADFGHYGGLFIRMAWHSAGTYRVFDGRGGGGGGQQRFAPLNSWPDNVS : 125
VdaKatG1L   : NALKLNILRQHTDVTNPLGNDFDYAAAFNSLDYNAVKKDLKDLMTDSQDWWPADFGHYGGLFVRMAWHSAGTYRVFDGRGGGGQGQQRFAPLNSWPDNVS : 128
VaaKatG1    : NALKLNILRQHTDVTNPLGSDFDYAAAFNSLDYNAVKKDLKDLMTDSQDWWPADFGHYGGLFVRMAWHSAGTYRVFDGRGGGGQGQQRFAPLNSWPDNVS : 128
VdaKatG1V   : NALKLNILRQHTDVTNPLGSDFDYAAAFNSLDYNAVKKDLRDLMTDSQDWWPADFGHYGGLFVRMAWHSAGTYRVFDGRGGGGQGQQRFAPLNSWPDNVS : 128
CgraKatG1   : NSLRLNILRQHTPVTNPLGGDFDYVSAFKGLDYDAIKKDLTALMTDSQEWWPADFGHYGGFFIRMAWHSAGTYRVHDGRGGGGEGQQRFAPLNSWPDNVS : 126
ChigKatG1   : NSLRLNILRQHTPVTNPLGGDFDYVSAFKGLDYDGIKKDLTALMTDSQEWWPADFGHYGGFFVRMAWHSAGTYRVHDGRGGGGEGQQRFAPLNSWPDNVS : 126
MacrKatG1   : ESVKLNVLRQNTPVTNPFGEEFDYAAAFKTLDYEALKKDLTALMTDSQEWWPADFGHYGGLFIRMAWHSAGTYRVFDGRGGGHAQQRFAPLNSWPDNVS : 123
PEchKatG1   : NSLKLGILRQHTDATNPQTKDFDYAAAFKTLDYWGLKKDLHALMTDSQEFWPADFGHYGGLFIRMAWHSAGTYRVFDGRGGGGQGQQRFAPLNSWPDNVS : 125
MagKatG2    : CQLRLDVLRQFQPSQNPLGGDFDYAEAFQSLDYEAVKKDIAALMTESQDWWPADFGNYGGLFVRMAWHSAGTYRAMDGRGGGMGQQRFAPLNSWPDNQN : 173
CgraKatG2   : CQLRLDVLRQFQPSTNPYGGDFDYVKAFSSLDYDALKADLRALLTESQDWWPADFGHYGGLFIRLCWHSAGTYRAIDGRGGGMGQQRFAPLNSWPDNQN : 167
                6 l 6LRQ       P     f1Y  AF   LDY   6KkD6   66T Sq 5WPADFGhYGGlF6R6aWHSAGTYRv DGRGGGg gQQRFAPLNSWPDNvs
```

```
                      *       220         *       240         *       260         *       280         *       300
CgKatG1    : LDKARRLLWPVKQKYGDKISWADLLLLTGNVALESMGFKTFGFAGGRPDTWEADESAYWGGEKTWL--GNDVRYGQGNEGVAGQGVVDGDESKKGHRDIH : 221
CcochKatG1 : LDKARRLLWPVKQKYGDNISWADLLLLTGNVALESMGFKTFGFAGGRPDTWEADESAYWGGEKTWL--GNDVRYSHGNEGVAAGGVVDGDESKKGHRDIH : 221
MthKatG1   : LDKARRLLWPIKQKYGDKISWADLLLLTGNVALESMGFKTFGFAAGRPDVFEADESIYWGGEQTWL--GNDVRYSQGREGVAGQGALGGDESKKDHKDIH : 221
TterKatG1  : LDKARRLLWPIKQKYGDKISWADLLLLTGNVALESMGLKTFGFAGGRPDTWEADESTYWGGETTWL--GNDVRYSDGQAGIAGDGVTGGDESKKVNKDIH : 220
PanKatG1   : LDKARRLLWPIKQKYGNKISWADLMLLTGNVALESMGFKTFGFAGGRPDTWEADESAYWGGETTWL--GNEARYAHGQEGIAGKGIVSGDESKKNHTDIH : 224
CtheKatG1  : LDKARRLLWPVKQKYGDKISWADLMLLAGNVALESMGLKTFGFAGGRPDTWEADESAYWGGETTWL--GNEVRYAN--------------------KDIK : 201
MagKatG1   : LDKARRLLWPIKQKYGNKISWADLMLLTGNVALEDMGFKTFGFAGGRPDTWEADESTYWGGETTWL--GNEVRYSSGNEGHKESGVIDGSESKKGHKDIH : 221
SmaKatG1   : LDKARRLLWPIKQKYGNKISWADLLLLTGNVALESMGFKTFGFAGGRPDTWEADESTYWGSETTWL--GNEDRYAEGQEGPKGHGVVQGDESKKDHKDIH : 221
NtetKatG1  : LDKARRLLWPIKQKYGNKISWSDLLLLTGNVALESMGFKTFGFAGGRPDTWEADESVYWGAESTWL--GNEDRYSEGQEGHEGHGVVQGDESKKQHTDIH : 221
MgKatG1    : LDKARRLLWPIKQKYGNKISWADLFLLTGNVAIESMGLPTFGFAGGRADTWEADDSVYWG-ETTWL--GNEVRYSDGKEGLTGDGILDGDQSKKQHTDIH : 222
F2E518_Hv  : LDKARRLLWPIKQKYGNKISWADLMLLTGNVALESMGFKTFGFAGGRADQWEADESVYWGGEFTWL--GNEVRYANGKEGVSGHGVVDGDEHKKGHSDIH : 223
VdaKatG1L  : LDKARRLLWPIKQKYGNKISWADLLLLTGNVALESMDFKTFGFAGGRADVWEADESVYWGGETTWL--GNDVRYSGGNKGDKGPGSLVTDEG--HDKSTH : 224
VaaKatG1   : LDKARRLLWPIKQKYGNKISWADLLLLTGNVALESMDFKTFGFAGGRADVWEADESVYWGGETTWL--GNDVRYSGGNKGDKGPGSLVTDEG--HDKGTH : 224
VdaKatG1V  : LDKARRLLWPIKQKYGNKISWADLLLLTGNVALESMDFKTFGFAGGRADVWEADESVYWGGETTWL--GNDVRYSGGNKGDKGPGSLVTDEG--HDKGIH : 224
CgraKatG1  : LDKARRLLWPIKQKYGNKISWADLMILTGNVALESMGFKTAGFSGGRPDTWEADESVYWGGETTWL--GNDVRYSHGHRGKADSGVVDGSQS--TKSDIH : 222
ChigKatG1  : LDKARRLLWPIKQKYGNKISWADLMILTGNVALESMGFQTAGFSGGRSDTFEADESVYWGGETTWL--GNDVRYAHGHAGKADSGVLDGSQATKAKSDIH : 224
MacrKatG1  : VDKARRLLWPIKQKYGNKISWADLLILAGNVALESMGFKTYGFAGGRVDEWEADESVFWGGETTWL--GNDVRYND-------------------NKDVK : 202
PEchKatG1  : LDKARRLLWPIKQKYGNKISWADLLLLTGNVALESMGFKTFGFAGGRPDVWEADESVYWGSENVWF--TNKARYEAETAEEEAK-----------QGNIK : 212
MagKatG2   : LDKARRLIWPIKQKYGNKISWADLMLLTGNVALENMGFKTLGFGGGRADTWQSDEAVYWGAETTFVPQGNDVRYNN-------------------SVDIN : 254
CgraKatG2  : LDKARRLLWPIKQKYGNKISWADLILLAGNTALDDMGFKTLGFAAGRPDTWQADESIYWGGETTFVPKGNDVRYNG-------------------STDIF : 248
             6DKARRL6WP6KQKYG1kISWaDL 6LtGNvA6esMgfkT GFagGR D 52aDes 5WG E t5   gN vRY
```

```
                       *       320         *       340         *       360         *       380         *       400
CgKatG1     : SRD--LESPLAAAHMGLIYVNPEGPDGIPDPVAAGRDIRTTFGRMAMNDEETVALIAGGHTFGKTHGAAPAVNVSKEPEAAPLEQQGLGWSNKHGTGKGP : 319
CcochKatG1  : SRD--LESPLAAAHMGLIYVNPEGPDGIPDPVAAGRDIRTTFGRMAMNDEETVALIAGGHTFGKTHGAAPAVNVSKEPEAAPLEQQGLGWSNKHGSGKGP : 319
MthKatG1    : TRD--LETPLAASHMGLIYVNPEGPDGIPDPLAAARDIRITFGRMAMNDEETVALIAGGHTFGKTHGAAHSDNVGPEPEAAPLEQQGLGWSNKHGSGKGP : 319
TterKatG1   : SRD--LESPLAAAHMGLIYVNPEGPDGIPDPVAAARDIRTTFSRMAMNDEETVALIAGGHTFGKTHGAAPSDNVGKEPEGAGLEQQGLGWANKHGSGKGP : 318
PanKatG1    : NRD--LESPLAAAHMGLIYVNPEGPDGNPDPVAAARDIRVTFGRMAMDDEETVALIAGGHTFGKTHGAAPADNVGAEPEAASIEQQGFGWSNKYGSGKGP : 322
CtheKatG1   : NRE--LETPLAAAHMGLIYVNPEGPDGNPDPVAAARDIRITFGRMAMNDEETVALIAGGHTFGKTHGAGPTDNVGKEPEAAPIEEQGFGWSNKFGSGKGP : 299
MagKatG1    : TRD--LEKPVSAAHMGLIYVNPEGPDGIPDPVAAARDIRTTFSRMAMNDEETVALIAGGHTVGKTHGAAPSDNVGPEPEAAPIENQGLGWSNKHGSGKGP : 319
SmaKatG1    : TRD--LQNPLAAAHMGLIYVNPEGPDGIPDPVASAKDIRVTFGRMAMNDEETVALIAGGHTFGKTHGAGPTHHVGKEPEAAPIEQQGLGWANSFGQGKGP : 319
NtetKatG1   : NRD--LQSPLASSHMGLIYVNPEGPDGIPDPVASAKDIRVTFGRMAMNDEETVALIAGGHSFGKTHGAGPTHHVGKEPEAAPIEHQGLGWANSFGQGKGP : 319
MgKatG1     : GRE--MEEPIAAAHMGLIYVNPEGPDGKPDPVASARDIRTTFGRMAMNDAETVALIAGGHAFGKTHGAGPTDNVGKEPNGASIEEQGLGWKNQYKDGKGP : 320
F2E518_Hv   : SRE--LEEPLAAVHMGLIYVNPEGPDGIPDTKASARDIRTTFGRMAMNDEETVALIAGGHTFGKTHGAGPSDNVGKEPEAAEIELQGFGWQNKFKSGKGP : 321
VdaKatG1L   : TRG--LQKPLGAAHMGLIYVNPEGPDGNPDPVAAAHDIRTTFSRMAMNDEETVALIAGGHSFGKTHGAGPNDKIGAEPEGASLEAQGFGWQNGYKSGKGP : 322
VaaKatG1    : TRD--LQKPLGAAHMGLIYVNPEGPDGNPDPVAAAHDIRTTFGRMAMNDEETVALIAGGHSFGKTHGAGPNDKIGAEPEGASLEAQGFGWQNGYKSGKGP : 322
VdaKatG1V   : TRD--LQKPLGAAHMGLIYVNPEGPDGNPDPVAAAHDIRTTFGRMAMNDEETVALIAGGHSFGKTHGAGPNDKIGAEPEGAPLEAQGFGWQNGYKSGKGP : 322
CgraKatG1   : SRD--LESPLGAAHMGLIYVNPEGPDGNPDPVAAARDIRITFGRMAMNDEETVALIAGGHSFGKTHGAAPDSNVGPEPEAAGLEAQGLGWHNKHGSGKGP : 320
ChigKatG1   : SRE--LESPLGAAHMGLIYVNPEGPDGNPDPVAAARDIRTTFGRMAMNDEETVALIAGGHSFGKTHGAAPDSNVGPEPEGAGLEQQGLGWVNKHGSGKGP : 322
MacrKatG1   : KRD--LESPLAASHMGLIYVNPEGPDGNPDPVAAARDIRTTFGRMAMNDEETVALIAGGHTFGKTHGAGPATNVGKEPEGAPIEQQGLGWKSTYGTGKGP : 300
PEchKatG1   : TRD--LEDPLAAVVMGLIYVNPEGPDGNGDPLSAARDIRITFGRMAMNDEETVALIAGGHTFGKTHGAAPADNVGTEPEAAGIESQGLGWHNKHGSGVGP : 310
MagKatG2    : ARADKLEKPLAATHMGLIYVNPEGPNGTPDPAASAKDIREAFGRMGMNDTETVALIAGGHAFGKTHGAVKGSNIGPAPEAADLGMQGLGWHNSVGDGNGP : 354
CgraKatG2   : ERADKLEKPLASTNMGLIYVNPEGPDGSSNPAASANDIRTAFHRMGMNDSETVALIAGGHAFGKTHGAVSADNIGPEPEAASIGEMGLGWHNSVGDGNGV : 348
               R   62 P6 a hMGLIYVNPEGP1G p1p a a DIR tFgRMaM1DeETVALIAGGH fGKTHGA p   6g ePe A 6e qG GW n    GkGp
```

```
                      *        420         *        440         *        460         *        480         *        500
CgKatG1    : DTITSGLEVIWTKEPTKWTHNFFEYLFKFEWELTKSPAGANQWVAKNTEPIIPDAFDPNKKHLPRMLTTDLALRFDPEYEKISRRFLENPDQ-FADAFAR : 418
CcochKatG1 : DTITSGLEVIWTKEPTKWTHNFFEYLFKFEWELTKSPAGANQWVAKNSEPIIPDAYDPNKKHLPRMLTTDLALRFDPEYEKISRRFLENPDQ-FADAFAR : 418
MthKatG1   : DTITSGLEVIWTKEPTKWNHNFFEYLFKFEWELTKSPAGANQWVAKDTDAFIPDPYDPNKKHKPRMLTTDLALRFDPVYEKISRRFLEHPDQ-FADAFAR : 418
TterKatG1  : DTITSGLEVIWTKTPTRWSTNFLEYLFKFEWELTKSPAGANQWVAKNADAFIPDAYDPSKKHRPRMLTTDLALRFDPVYEKIARHYLEHPDQ-FADAFAR : 417
PanKatG1   : DTITSGLEVIWTKNPTKWTNQFFEYLFKYEWELTKSPAGANQWVAKNAEPFIPDAYDPNKKHLPRMLTTDLSLRFDPGFEKISRRFLEHTDQ-FADAFAR : 421
CtheKatG1  : DTITSGLEVIWTKTPTQWSTNFLEYLFKFDWELTKSPAGANQWVAKNAEAFIPDPYDPSKKHPPRMLTTDLALRFDPVYEKISRRFLEHPDE-FADAFAR : 398
MagKatG1   : DTITSGLEVIWTKEPAKFTMNYLEYLFKYEWELTKSPAGANQWVAKNAEEFIPDAFDPSKKHKPRMLTTDLSLRFDPEYEKISRRFLENPEQ-FKDAFAR : 418
SmaKatG1   : DTITSGLEVTWTPTPTKWGMGYLEYLYKFEWEQTKSPAGANQWVAKNAEPSIPDAYDPNKKKLPTMLTTDLALRMDPAYDKICRDFLANPNK-FADAFAR : 418
NtetKatG1  : DTITSGLEVTWTPTPTKWGMGYLEYLYKFDWEPTKSPAGANQWVAKNAEPTIPDAYDPNKKKLPTMLTTDIALRMDPAYDKICRDYLANPDK-FADAFAR : 418
MgKatG1    : NAITSGLEVIWTSTPTKWSNKYLEYMFKYEWELTKSPAGAHQWTAKTDDEIIPDAYDSTKKHKPTMLTSDLALRFDPEYEKISRDYLENPDK-LADAFTR : 419
F2E518_Hv  : DTITSGLEVTWTKTPTKWSNQYFEYLFKYEWELTKSPAGANQWVAKNAEPIIPHAYDANKKQLPTMLTTDIALIHDPEYRKISERFLKNPDQ-FADAFAR : 420
VdaKatG1L  : DTITSGLEVTWTATPTKWSNKYFEYLFKYEWELTKSPAGANQWVAKTDDEIIPDAYDSSKKHRPTMLTTDLSMRFDPEYEKISRRFLENPDQ-FADVFAR : 421
VaaKatG1   : DTITSGLEVTWTATPTKWSNKYFEYLFKYEWELTKSPAGANQWVAKTDDEIIPDAYDSSKKHRPTMLTTDLSMRFDPEYEKISRRFLENPDQFFADAFAR : 422
VdaKatG1V  : DTITSGLEVTWTATPTKWSNKYFEYLFKYEWELTKSPAGANQWVAKTDDEIIPDAYDSSKKHRPTMLTTDLSLRFDPEYEKISRRFLENPDQ-FADAFAR : 421
CgraKatG1  : DTITSGLEVTWTATPTKWSNKYFEYLFKFDWELTKSPAGANQWVAKNAEPIIPDAYDPNKKHLPTMLTTDLSLRFDPEYEKISRRFLENPDQ-FADAFAK : 419
ChigKatG1  : DTITSGLEVTWTATPTKWSNKYFEYLFKYDWELTKSPAGANQWVAKNAEPIIPHAYDPNKKQLPTMLTTDLSLRFDPEYEKISRRFLENPDQ-FADAFAK : 421
MacrKatG1  : DTITSGLEVIWTKTPVQWSHNYFQYLFKYEWELTKSPAGANQWVAKTDDEIIPDAFDANKKHKPRMLTTDLSLRFDPEYEKISRRFLENPDQ-FADAFAR : 399
PEchKatG1  : HAITSGLEVTWTSTPTKWSNSFLDYLFKFEWEQTKSPAGATQWVAKNAGDIVPDAFDSSKKHRPHMLTTDLSLRYDPAYEKISRRFLENPDE-FADAFAR : 409
MagKatG2   : NQMTSGLEVIWTKTPTKWSNGYLESLINNNWTLVESPAGAHQWEAVNGTVDYPDPFDKTKFRKATMLTSDLALINDPEYLKISQRWLEHPEE-LADAFAK : 453
CgraKatG2  : NQMTSGLEVIWTKTPTKWSNDYLESLLHNKWTLVTSPAGHHQWEAVNGTLDYPDPFDATKFRKATMLTSDLALINDPSYLNITTRWVDHPDE-LADAFAK : 447
              t6TSGLEV WT  Pt 5   5  y6 k  WeltkSPAGanQWvAk      Pda5D  Kk  p MLT3D6 6r DP 5 kI rr56e p   faDaFa4
```

```
                        *         520         *         540         *         560         *         580         *         600
CgKatG1     : AWFKLLHRDLGPRSRWLGPEIPSEVLIWEDPVPAVNHPLVDEQDVTTLKRAILAT-GVAPAKLISTAWASASTFRGGDKRGGANGARIRLAPQKDWKVNN : 517
CcochKatG1  : AWFKLLHRDLGPRSRWLGPEIPAEVLLWEDPVPAVNHPLVDDQDVTALKRDILAT-GIAPANLISTAWASASTFRGGDKRGGANGARIRLAPQKDWEVNN : 517
MthKatG1    : AWFKLLHRDMGPRSRWLGPEIPAEVLPWEDPIPPVNHPLVDDKDIAALKHDILAT-GVAPHKLISTAWASASTFRGSDKRGGANGARIRLAPQKDWEVNN : 517
TterKatG1   : AWFKLLHRDMGPRSRWLGPEIPSEVLLWEDYVPPVDHPLVDDSDIAALKRDILAT-GVAPAKLISVAWASASTFRGSDKRGGANGARIRLAPQKDWKVNN : 516
PanKatG1    : AWFKLLHRDMGPRSRWLGPEIPSEVLLWEDPLPPLDHPVIDNNDIAAIKREILAT-GLAPQKLISTAWASASTFRGSDKRGGANGARIRLAPQKDWKVNN : 520
CtheKatG1   : AWFKLLHRDMGPRTRWLGPEIPSEILPWEDYIPPVDYPVIDDADVAALKQTILAT-GIAPHKLISTAWASASTFRGSDKRGGANGARIRLAPQKDWPVNN : 497
MagKatG1    : AWFKLLHRDMGPRSRWLGPEVPKETLLWEDPIPTPDHPIIDGSDVDSLKKAILAT-GVAPSKLIQTAWASASTFRGGDKRGGANGARIRLEPQNKWEVNN : 517
SmaKatG1    : AWFKLLHRDMGPRTRWIGPEVPSEVLPWEDYIPPVDYQVIDDNDIKLLKKEILAT-GVAPKKLIFVAWSSASTFRGSDKRGGANGARIRLAPQNEWKVND : 517
NtetKatG1   : AWFKLLHRDMGPRTRWIGPEVPSEILPWEDYIPPVDYQIIDDNDIAALKKEIMAT-GVAPKKLIFVAWSSASSFRGSDKRGGANGARIRLAPQNEWKVND : 517
MgKatG1     : AWFKLLHRDMGPRSRWVGPEIPKEVLIWEDPVPSVNHPLVDEKDVAALKKAIIAT-GVEPTALISAAWASASSYRGSDKRGGANGARVRLEPQKDWKVNN : 518
F2E518_Hv   : AWFKLLHRDLGPRSRWVGPELPSEVLLWEDPVPAVDHPLIDEKDAASLKKEILAT-GVEPTKLIRTAWASASTFRGSDKRGGANGARIRLAPQKDWKANN : 519
VdaKatG1L   : AWFKLLHRDLGPKARYLGPEIPAEDLLWQDPIPAVDHPLIDESDIAALKKEILSS-GPEPSQFISVAWGASSTFRGSDKRGGANGARIRLAPQKDWEVNN : 520
VaaKatG1    : AWFKLLHRDLGPKARYLGPEIPAEDLLWQDPIPAVDHPLIDESDIAALKKEILSS-GPEPSQFISLVWGASSFFSRQRQRGDVNGARIRLALQKDWEVYI : 521
VdaKatG1V   : AWFKLLHRDLGPKARYLGPEIPAEDLLWQDPIPAVDHPLIDESDVAALKKEILSS-GPEPSQFISVAWGASSTFRGSDKRGGANGARIRLAPQKDWEVNN : 520
CgraKatG1   : AWFKLTHRDMGPRSRYVGPEVPSEEFIWQDPIPTPNHPLVNEQDAASLKKEILGT-GIDPSKLISTAWASASTFRGSDKRGGANGARIRLAPQKDWEVNN : 518
ChigKatG1   : AWFKLTHRDMGPRSRYVGPEVPSEDFIWQDPIPAPNHPLINEQDAASLKKEILGT-GIDPSKLVSTAWASASTFRGSDKRGGANGARIRLAPQKDWEVNN : 520
MacrKatG1   : AWFKLTHRDMGPKTLYVGPEVPTEDLIWQDPIPPVNYQLIDDKDAAAIKEEILKS-GIDTSKLVSTAWASASTFRGSDKRGGANGARIRLSPQKDWKVNN : 498
PEchKatG1   : AWFKLLHRDMGPRARWLGPEIPKEVSLWEDPIPAPTYALIDNGDIIALKNEILSA-GIEPTKLIATAWASASTFRGGDKRGGANGAHIRLAPQKDWDVNN : 508
MagKatG2    : AWFKLLHRDLGPTTRYLGPEVPKESFIWQDPLPAREGDLIDDADVDKLKAAILSTDGLDVSKLASTAMACATTYRNSDKRGGCNGARIALEPQRNWVSNN : 553
CgraKatG2   : AWFKLLHRDMGPRARYLGPEVPQETFIWQDPLPAREGDLMDDADVATLKSTILAVPGLDVSKLASVAWASASTFRGSDKRGGANGARIALEPQVNWVANN : 547
              AWFKLlHRD6GP  r56GPE6P E   W2Dp6P     661  D   6K  I6    G  p  l   aw sa3 5rg dkRGgaNGAr6rL pQ  W vn
```

```
                       *       620         *       640         *       660         *       680         *       700
CgKatG1     : PPQLAEVLKALEGVQAQFNGASQS-KKVSLADLIVLGGVAALEQAAG-----VSVPFTPGRTDASQEQTEVESFAHLEPHVDGFRSYGRGTSRVSTEQFL : 611
CcochKatG1  : PTQLAEVLKALEGVQAKFNGAAQGGKKVSLADLIVLGGVAALEQAAG-----IAVPFTPGRTDASQEQTEVESFAHLEPHVDGFRSYGRGTSRVSTEQFL : 612
MthKatG1    : PPQLAEVLKALEGVQAAFNASAPGGKKVSLADLIVLGGVAALEQAAG-----VPVPFSPGRTDASQEQTDVESFQHLKPFADGFRNYGRGTSRVTTEQLL : 612
TterKatG1   : PPQLAEVLQALEGVQAKFNASAAGGKKVSLADLIVLGGVAALEQAAG-----VPVPFRPGRTDASQEQTDVASFAHLEPYADGFRNYGRGTARVPTEHLL : 611
PanKatG1    : PAQLAEVLGALEDVQKRFNEQATGGKKVSLADVIVLGGVAALEQAAG-----VSVPFTPGRTDASQEQTDVHSFEHLEPYADGFRNYGHGNDRVKTEQYL : 615
CtheKatG1   : PAQLAEVLSALEAVQAQFNSTAPGGKKVSLADLIVLGGVAALEQAAG-----VPVPFTPGRNDTTQEYTEVESFSYLEPVADGFRNYGRGTDRVRTEQLL : 592
MagKatG1    : PQQLAEVLKALEGVKADFE---KSGKKVSIADLIVLAGVAAVEQAAG-----VPVPFTPGRGDATQEQTDVESFTHLEPAADAFRNYGKGTSRVTTEQIM : 609
SmaKatG1    : PATLREVLSALESIQQKFNNS-SSGKKVSLADLIVLGGVAALEQASG-----LVVPFTPGRNDATQEQTDVHSFGHLEPYADGFRSYGKGTERVRTEQFL : 611
NtetKatG1   : PSTLREVLAALESVQQKFNDS-SSGKKVSLADLIVLGGVAALEQASG-----LVVPFTPGRNDATQEQTDVHSFTHLEPHADGFRSYGKGTKRVRTEQFL : 611
MgKatG1     : PSQLSKTLSALEGVQKDFNAS-SGSKKVSLADLIVLAGSAGIEKTSG-----VHVPFTPGRTDATQEQTDAQSFTHLEPKVDGFRNYGKGTERVRAEQCL : 612
F2E518_Hv   : PTDLAEVLKALEGVQQKFNS---GSKKVSLADLIILAGNAAIEKASG-----ASVPFTPGRTDATEEQTDAQSFEHLEPVVDAFRNYGKGTKRVRTEQFF : 611
VdaKatG1L   : PAQLAKVLQALEGVQKSFNDAQQGGKKVSLADLIVLAGNAAVEKAASAAGHSVTVPFTPGRGDATQEQTDIESVSHLEPFADGFRNYGHSTDRVKTEQFL : 620
VaaKatG1    : ARPAVQGMQALEGXX------------------------------HS-----VTVPFTPGRGDATQEQTDIESVSHLEPFADXFRNYGHSTDRVKTEQFL : 586
VdaKatG1V   : PAQLAKVLQALEGVQKSFNDAQQGGKKVSLADLIVLAGNAAVEKAASAAGHSVTVPFTPGRGDATQEQTDIESVSHLEPFADGFRNYGHSTDRVKTEQFL : 620
CgraKatG1   : PRQLAEVLKALESVQQKFNSSASGGKKISLADLIVLAGAAGVEQAARNAGYDITVPFTPGRGDASQEQTDIESVDHLQPFADGFRNYGKSTDRAKTEHYL : 618
ChigKatG1   : PRQLAEVLKALEGVQQKFNSSSSGGKKVSLADLIVLAGAAGVEQAAKNAGYDITVPFTPGRGDASQEQTDVESIDHLQPFADGFRNYGKSTDRAKTEHYL : 620
MacrKatG1   : PAQLSQVLATLEDIQKRFNQS---GKTVSLADVIVLAGVAGVEKAARDAGVNITVPFTPGRNDATQEQTDVESVGYLEPLADGFRNYGKSHGSVKQEHLL : 595
PEchKatG1   : PAQLQEVLGLLESIQGRFNKAQNGEKRVSIADLIVLAGSAALERAAG-----IPVPFTPGRNDATQEQTDVESFGWLRPFADGFRNYGNSTRRVRTEQLL : 603
MagKatG2    : PTQLSAVLDALKKVQSDFNGS-NGNKKVSLADLIVLGGTAAVEKAAKDAGVDIKVPFSAGRVDATQEQTDVTQFSYLEPQADGFRNYGRGTARARTEEIM : 652
CgraKatG2   : PTQLSAVLDALKKVQADFN---TGSKKVSLADLIVLGGTAAVEKAAADAGVKVTVPFAPGRVDATQDQTDIESFGYLEPQADGFRNYGRGTARARTEEFL : 644
              p ql  v6 aLe  q  fn      k  s ad i l g a  e a         VPF pGR Da32eqTd  s   L P aDgFRnYG  t rv tE
```

```
                          *       720         *       740         *       760         *       780         *       800
CgKatG1      : VDRAHLLTLTPPELAVLVGGLRVLGANYDGSSNGVFTTRPGKLTNDYFVNLLDMATAWKSVDG--EVFEGNDRKTGEKKWTGTRADLVFGAHAELRAISE : 709
CcochKatG1   : VDRAHLLTLTPPELAVLIGGLRVLGANYDGSSNGVFTTRPGKLTNDYFVNLLDMGTAWKSVDG--EVFEGSDRKTGEKKWTGTRADLVFGAHAELRAISE : 710
MthKatG1     : VDRAQLLTLTPPELTVLVGGLRVLGANYDGSSHGVFTTRPGKLTNDFFVNLLDTGVAWKSIDG--ELFEGTDRKTGEKKWTGTRADLIFGAHAELRAIAE : 710
TterKatG1    : VDRAQLLTLTPPELTVLVGGLRVLGANYDGSSHGVFTTRPGKLTNDFFVNLLDMRTAWKSVDG--ELFEGTDRATGAKKWTATRADLVFGSHAELRAIAE : 709
PanKatG1     : VDRAHLLTLTAPELAVLVGGLRVLGANYDGSDHGVFTAQPGKLTNDFFVNLLDPNTEWTNVDGKGEVFEGKDRATGQKKWTGTRADLIFGSHSELRAIAE : 715
CtheKatG1    : VDRAHLLTLTPPEMTVLVGGLRVLGANWDGSTHGVFTKRPGQLTNDFFVNLLDMSTAWRSIDG--EIFEGYDRKTGEKKWTATRVDLIFGSHAELRAIAE : 690
MagKatG1     : VDRAQQLTLTAPELTVLVGGLRVLGANYDGSSHGVWTDKPGKLTNDFFVTLLDPYTSWKSVDG--EVFEGTNSKSG-KKLTGTRADLVFGSHSELRALAE : 706
SmaKatG1     : IDRASLLTLSAPEFTALIGGLRVLEANYDGGSHGVLTKTPGKLTNDFFVNLLDTNTAWKKADNEGELFIGYDRKTHDKKWTATRADLIFGSHAELRALAE : 711
NtetKatG1    : IDRASLLTLSAPELTALIGGLRVLEANYDGSSYGVLTKTPGKLTNDFFVNLLDTNTAWKAADNEGEVFIGYDRKTHDKKWTATRADLIFGAHAELRALAE : 711
MgKatG1      : IDKAHLLTLTAPEMTALVGGLRSLNQNFDGSKHGVFTERPGVLSNDFFVNLLDSNIDWKATGD--ETYEGVERSSGQKKWTATRHDLVFGSHPELRAVSE : 710
F2E518_Hv    : VDKAHLLTLTAPEATVLVGGLRALNANWDGSSYGVFTQRPGQLTNDFFINLLDINTAWKASDGSNEVFEGGDRKTGSKKWTATRHDLIFGSHPELRAIAE : 711
VdaKatG1L    : VDRAHLLTLSAAELTALVGGLRVLNTNYDGSQHGVLTKRPGQLSNDFFVNLLDMSTAWKATGSDDELFEGSDRKTGDKRWTATRADLVFGSHAELRALAE : 720
VaaKatG1     : VDRAHLLTLSAPELTALVGGLRVLNTNYDGSQHGVLTKRPGQLSNDFFVNLLDMSTAWKATGSDDELFEGSDRKTGDKRWTATRADLVFGSHAELRALAE : 686
VdaKatG1V    : VDRAHLLTLSAPELTALVGGLRVLNTNYDGSQHGVLTKRPGQLSNDFFVNLLDMSTAWKATGSDDELFEGSDRKTGDKRWTATRADLVFGSHAELRALAE : 720
CgraKatG1    : VDRAHLLTLTAPELTVLLGGLRVLNTNFDGSSTGVFTSRPGALTTDFFVNLLDMGNEWKATSN-EDVYEGYDRKSGSKKFTASRVDLIFGSHAELRAVAE : 717
ChigKatG1    : VDRAHLLTLTAPELTVLLGGLRVLNTNFDGSSTGVFTNRPGALTNDFFVNLLDMGNEWKATNN-QDVYEGYDRKSGSKKYTASRVDLIFGSHAELRAVAE : 719
MacrKatG1    : VDRAHLLTLSAPELAVLVGGLRALNANYDGSALGVFTSRPGVLSNDFFVNLLDNNTEWRATG--EDTFEGFDRKTGAKKWIASRNDLIFGAHPELRAVSE : 693
PEchKatG1    : IDKAQQLTLSAPELTVLLGGLRSLNANWDGSSHGVFTSRPGQLSNDFFVNLLDMSTVWKPTDADSEKFEGFDRKTGAKKWSATRVDLIFGHHAELRALSE : 703
MagKatG2     : VDKASQLTLTPPELTVLVGGMRALGANYDGSDVGVFTANKGKLTPDFFVNLVDMNIAWTASGADGESWVGTDRKSRSEKYKGSRADLVFGSHAELRAIAE : 752
CgraKatG2    : VDKAAQLTLSPPEMTVLVGGLRALGAVFDGSNTGVLTEKKGQLTNDYFKNLLDISTVWSQSDTDGETWKGVDRKTKTEKWTATRADLVFGSHAELRAISE : 744
               6D4A lLTL3 pE t L6GG6R L  n5DGs  GV T  pG L3nD5F nL6D    W       e 5eG  rk3g k4   3R DL6FG H ELRA6 E
```

PTS 1

```
                          *       820         *       840
CgKatG1     : VYGSSSGQDKFVKDFVAAWSKVMNLDRYDL----AQPGSSGPKL : 749
CcochKatG1  : VYGSSSGQDKFVKDFVAAWNKVMNLDRYDL----AQPGSSGPKL : 750
MthKatG1    : VYASAGGQDKFVKDFVAAWAKVMDLDRFDL----AQQGSAGPKL : 750
TterKatG1   : VYASAGGQEKFVKDFVAAWDKVMNLDRFDL----A--------- : 740
PanKatG1    : VYGSADGQEKFVKDFVAAWDKVMNLDRFDL----EQGAGSSPKL : 755
CtheKatG1   : VYASADGQEKFVRDFIAAWDKVMNLDRFDL----------GKR- : 723
MagKatG1    : VYGSADGQQKFTKDFVAAWDKVMNLDRFDVRRGIYDETRLKSKL : 750
SmaKatG1    : VYASVDGEDKFKRDFVAAWHKVMNLDRFDLR--QEGRGQNTPKL : 753
NtetKatG1   : VYAAVDGEEKFKRDFVAAWHKVMNLDRFDLK--QEGRGQNAPKL : 753
MgKatG1     : VYGSSDGADKFTRDFVSAWEKVMNADRFDLK----AGQVSSAKL : 750
F2E518_Hv   : VYGSSDAQDKFVKDFVAAWTKVTNLDRFDL---SSSASSSKARL : 752
VdaKatG1L   : VYGSADGEKKFVNDFVAAWTKVSNLDRFDVK--KAPAVKTSSRL : 762
VaaKatG1    : VYGSADAEKKFVNDFVAAWTKVSNLDRFDVK--KAPAVKTSSRL : 728
VdaKatG1V   : VYGSADGEKKFVNDFVAAWTKVSNLDRFDVK--KAPAVKTSSRL : 762
CgraKatG1   : IYAQADAGQKFVNDFVTTWNKVMNLDRFDLK---NNSGSVASRL : 758
ChigKatG1   : IYAQADAGQKFVKDFVTIWNKVMNLDRFDLKS---NSGSVPSRL : 760
MacrKatG1   : VYGSADGAQKFVKDFVAAWDKVMNLDRFDLK--R-GGNKKAARL : 734
PEchKatG1   : LYASSDAQDKFKKDFVAAWDKVMNLDRYDI---PCFPATGRPRL : 744
MagKatG2    : VYAENGNQEKFVKDFVAAWTKVMNLDRFDLKVKK---------- : 786
CgraKatG2   : VYASSDANEKFVNDFVAAWTKVMNLDRFDL--QK---------- : 776
              6Y s     KF  DF6aaW KVm1lDR5D6             l
```
